# Habit formation in support of antiretroviral medication adherence in clinic-enrolled HIV-infected adults: a qualitative assessment using free-listing and unstructured interviewing in Kampala, Uganda

**DOI:** 10.1186/s12981-020-00283-2

**Published:** 2020-06-08

**Authors:** Larissa Jennings Mayo-Wilson, Bianca Devoto, Jessica Coleman, Barbara Mukasa, Angela Shelton, Sarah MacCarthy, Uzaib Saya, Harriet Chemusto, Sebastian Linnemayr

**Affiliations:** 1 Indiana University School of Public Health, Department of Applied Health Science, Center for Sexual Health Promotion, 1025 E. 7th Street, Bloomington, IN USA; 2 Johns Hopkins Bloomberg School of Public Health, Department of International Health, Social and Behavioral Interventions Program, 615 N. Wolfe Street, Baltimore, MD USA; 3 Mildmay Uganda, Mildmay Hospital and Institute of Health Sciences, Box 24985, Kampala, Uganda; 4 RAND Corporation, 1776 Main Street, Santa Monica, CA USA

**Keywords:** Habit formation, Adherence, Antiretroviral therapy, ART, HIV, Qualitative, Uganda

## Abstract

**Background:**

Despite initial high motivation, individuals receiving antiretroviral therapy (ART) for several years may experience incomplete adherence over time, increasing their risk of HIV-related morbidity and mortality. Habits, defined as automatic and regular practices, do not rely on conscious effort, and may therefore support high long-term ART adherence.

**Methods:**

This qualitative study contributes to the evidence on how clients with adherence problems remember and form habits to take ART medications. Free-listing and unstructured interviewing were used among 42 clinic-enrolled adults in Kampala, Uganda who were receiving ART and participating in a randomized clinical trial for treatment adherence (clinicaltrials.gov: NCT03494777). Data were coded and analyzed using inductive content analysis.

**Results:**

Findings indicated that clients’ most routine habits (eating, bathing, sleeping) did not always occur at the same time or place, making it difficult to reliably link to pill-taking times. Efforts to improve ART habits included having a relative to ask about pill-taking, re-packaging medications, leaving medications in view, using alarms, carrying water, or linking pill-taking to radio/prayer schedules. Reported challenges were adhering to ART schedules during changing employment hours, social activities, and travel.

**Conclusion:**

While habit-forming interventions have the potential to improve ART adherence, targeting treatment-mature clients’ existing routines may be crucial in this population.

## Introduction

An estimated 23.3 million people globally were receiving antiretroviral therapy (ART) in 2018, with the greatest increase in coverage occurring in sub-Saharan Africa [1, 2]. Of all persons living with HIV in Africa, approximately 67% (~ 13.8 million) had obtained antiretroviral therapy in 2018 [1, 2]. While this represents a significant achievement in the scale-up of HIV treatment, research has shown that not all ART clients achieve adherence sufficient for viral suppression [3]. Achieving and maintaining viral suppression is critical for persons living with HIV because it prevents the transmission of the virus to others, slows down disease progression, and ultimately reduces the risk of an AIDS-related illness or death. Yet, data from clinic-based adult populations in sub-Saharan Africa suggest that between 21 and 44% of ART clients have poor adherence over time with their ART medications, defined as intake less than 90% [3, 4].

Reasons for poor ART adherence can be intentional, such as clients actively deciding not to follow their ART medication regimen due to intolerance [5–7], cost [6, 8], or stigma [5, 6, 8, 9]. In these cases, clients may consciously alter the dose, timing, or frequency of their ART medications. Poor adherence may also be in response to non-intentional and unconscious reasons such as forgetting due to distractions, lack of attention, or in the absence of cues or reminders [5, 10]. Clients who have been on ART for several years may also fail to take their pills due to treatment fatigue and waning motivation from engaging in mundane, daily pill-taking over their lifetime. For this reason, there is increasing interest in understanding how ART clients remember and form habits relating to medication adherence, as habits may offer the possibility for those with low motivation to continue to show high adherence.

A habit is defined as a “regular practice that tends to occur subconsciously” without a person’s thinking about it [11–13]. Habits are generally viewed as behaviors prompted by situational cues (i.e. time of day or a specific location) that lead to frequent repetition [11–13]. In turn, repetitive performances gradually shift an individual’s cognitive controls from deliberate to automatic processes [11–14]. A person can both form a habit of taking their medications (“medication habit”) in addition to relying on non-medication habits (i.e. wake time, meals, exercise routine) to remain conscious of their pill times. However, much of the research to-date examines habit formation for medication adherence relating to diet [15], oral contraception [16], and chronic disease management (i.e., diabetes, hypertension) [17–21]. Although ART requires repeated and consistent medication intake, there is a dearth of literature examining client habits as they relate to taking ART medications for HIV [22]. Studies of determinants of ART adherence have also tended to focus on differences in demographic characteristics, treatment burden, or motivational factors, rather than automatic processes such as habits [5–9]. Therefore, less is known about whether and how clients rely on habits to maintain ART adherence, particularly those who have experienced recent adherence problems. In addition, although the literature on habit formation and medication adherence is growing, it has primarily investigated statistical associations within non-HIV populations and in American and European countries [15–18]. There is scarce qualitative exploration of what habits clients view as helping or hindering them to achieve the ART adherence they want, particularly in sub-Saharan Africa. Therefore, this study is among the first to qualitatively examine habit formation for ART adherence with treatment-mature adults in an African clinical setting. Specifically, we aimed to characterize the daily habits of ART clients who had recently missed doses and to understand how they described the process of linking to, creating, and/or changing habits to automate their ART pill-taking. Findings are to be used to inform future programming for support of clinic-based clients receiving ART.

## Methods

### Design

The data underlying this study were collected as part of the baseline qualitative assessment for HIV-positive adults who were newly enrolled in a randomized controlled trial (RCT) called “Behavioral Economics Incentives to Support HIV Treatment Adherence” (BEST) (clinicaltrials.gov: NCT03494777). The subsequent two-year trial will test the efficacy of small lottery incentives to support ART adherence for treatment-mature HIV clients. The cross-sectional baseline data were collected from April to July 2018.

### Sample recruitment

Eligible clients were recruited from a clinic in Kampala, Uganda that specialized in the provision of comprehensive HIV/AIDS prevention, care, and treatment services. Inclusion criteria were based on enrollment eligibility for the parent RCT and were: age 18 and older, receiving ART at the participating clinic for 2 or more years, and having demonstrated recent adherence problems defined as having either missed a clinic visit, obtained a prescription refill late within the past 6 months, been sent to adherence counseling, or lacking of viral suppression. Individuals were excluded if they were not mentally fit enough to provide informed consent, spoke neither English nor Luganda (the local language), were participating in any other adherence-related study, were using a third-line treatment, and/or were not capable of regularly using the trial’s upcoming MEMS-cap. The study team identified eligible clients using the clinic’s database. We then used the client’s date of next appointment to identify a date for potential recruitment into the study. On scheduled appointment dates, the study coordinator approached all eligible clients and invited them to participate in the study. Clients who were interested in the study were taken to a private room to verify eligibility and undergo informed consent. Enrolled clients were then provided a MEMS-cap and instructed on how to store their ART medications during the study period. During this enrollment session, a subset of ART clients enrolled in the parent RCT was also administered a qualitative interview in April and May 2018 prior to study group assignment. The target sample size for qualitative subset was 40 ART clients as this was expected to be sufficient to achieve saturation. The qualitative subset was chosen using a convenience sample of the first 40 clients to be enrolled in the parent RCT. A follow-up baseline interview to quantitatively assess demographic and self-reported adherence was conducted for all enrolled clients in the parent RCT in July 2018.

### Data collection

All qualitative interviews comprised of two methods: free-listing and unstructured interviewing. The interviews were conducted by two Ugandan interviewers who had prior experience conducting qualitative research relating to ART adherence. The interviewers underwent a 30-h training by the study team to familiarize themselves with the interview script and methodology. As part of the training, the interviewers conducted mock interviews with each other and test clients and met with the study team over the course of data collection to discuss interviewing goals and techniques.

Each interview began with a free-listing exercise to illicit a list of daily habits by the ART clients. Free-listing methodology was used to sample potential daily habits among clients without imposing investigator assumptions from a pre-coded survey [23–25]. Free-listing also provided the dual advantage of using open qualitative data collection that the study team could rapidly tally using quantitative methods [23–25]. The free-listing exercise began with the following primary statement to elicit a broad list of daily activities. Clients were told, “I’d like to learn about activities that people do in their daily life. This means things that they do every day regardless of whether it is a workday or a weekend.” We then asked each client to free-list “the activities that (s) he did without thinking every day” in the morning and in the evening. Relevant to the definition of a habit, this question aimed to assess clients’ regular practices that tended to occur subconsciously. Interviewers were trained to probe for as many activities as possible, asking for a brief description of each, as needed. The interviewer then created a written free-list of all activities mentioned by the client using a paper-based interview guide. When clients could think of no more activities, the interviewer returned to the top of the free-list and asked, “Do you do [specific activity mentioned] at the same time every day?” and “Do you do [specific activity mentioned] in the same location every day?” Asking clients about the consistency of timing and location of their activity aimed to provide context into the extent of their habitual behaviors. Based on the client’s response, the interviewer wrote “yes” or “no” in a tabular format adjacent to the free-list. If a client was not at ease with listing habits and instead narrated his/her routine, the interviewer then recorded ad hoc his/her responses to the list with time and location categories [26]. Pile sorts, which are sometimes used after free-listing, were omitted in this study because the lists were documented on a single form by the interviewer and did not include substantial variation [23–25].

The second qualitative method followed immediately after the free-listing exercise and involved an unstructured discussion. Clients were asked to share any habits or techniques that helped them to remember to take their ART medications. This question aimed to assess whether and how ART medication adherence was prompted by situational cues (i.e. time of day or a specific location) that led to frequent repetition. The interviewer probed on activities mentioned in the preceding free-list or any activities the client considered as “secrets” or “tips” to regularly taking their ART pills. Where applicable, clients were encouraged to describe habits linked to ART pill-taking (i.e., non-medication habits) as well as the potential habit itself of taking ART medications (i.e., medication habit). The entire interview was conducted in Luganda or English. We also obtained demographic and self-reported adherence data on enrolled clients (i.e. age, gender, highest level of education, marital status, employment status, years since HIV diagnosis, HIV disclosure status, missed ART doses in last month, stopped ART for 2 or more days, and currently has undetectable viral load) during a follow-up baseline interview in July 2018. In 5 cases (12%), these data were not obtained due to non-participation in the follow-up baseline interview. All 42 qualitative clients provided written informed consent prior to the interview. The approximate duration of each interview was 40 min. All interviews were audio-recorded, transcribed, and translated (if needed) into English. All clients received 20,000 Ugandan Shillings (equivalent to $5 USD) for their participation.

### Analysis

We generated 42 free-lists and 42 discussion transcripts from participating clients. The free-list data were analyzed in three phases. First, two research associates (RAs) met with the first-author investigator to develop one summary free-list that consolidated synonymous terms into distinct cover terms and included all of the different cover terms [26]. This was done in order to standardize the language of the analysis. For example, if one client mentioned “make tea” and another client mentioned “cook breakfast”, we created a cover term to include “make breakfast/tea”. The summary list consisted of 47 cover terms (referred to as “habits”). Habits mentioned by more than one client were listed only once. Next, we entered the summary list into a study-generated Excel database and coded whether each client mentioned the habit or not (“yes” code = 1 or “no” code = 0). We then calculated the number and percentage of clients who mentioned each habit as well as the number and percentage of clients who performed the habit at the same time each day or at the same location (“yes” code = 1 or “no” code = 0). If a client did not mention the habit on his/her individual free-list, we entered “not applicable” (code = 99) for the two timing and location questions. If a client mentioned the habit on his/her individual free-list but provided no response to the same time and same location questions, we entered “missing” (code = 88). All frequencies were then tallied. As a third and final step, we analyzed the free-list data using category construction informed by grounded theory [27]. This entailed using an iterative process reviewing the summary free-list and identifying emerging categories of similar habits. We identified five categories to represent related types of habits. They were: “eating habits” that relating to preparing and consuming meals; “domestic habits” relating to cleaning and other duties in one’s home; “leisure habits” such as sleeping or visiting friends; “hygienic habits” such as bathing and dressing; and “medication habits” relating to taking ART pills. We then reviewed the most commonly reported habits (mentioned by > 10% clients) within each of the five categories to preliminarily explore differences in the consistency of types of habits for ART client.

The discussion transcripts were also analyzed using a three-step process. We used an inductive content analysis methodology, a technique used in qualitative research to categorize verbal data based on emergent themes instead of categorizing based on previously structured topics or hypotheses [28, 29]. As part of this process, we first developed a provisional codebook based on a close reading of all of the transcripts. New codes were created and revised based on reading the transcripts, discussing codes within the study team, and finalizing changes based on a test of the provisional codebook on five of the 42 transcripts (> 10%). The provisional test of the codebook on a sample of transcripts additionally enabled the study’s two RAs to harmonize their coding techniques. Next, a finalized codebook was used to conduct line-by-line coding of all of the transcripts using ATLAS.ti, a qualitative data analysis software (Version 8.3.1, https://atlasti.com, Corvallis, OR, USA). Both RAs coded all 42 transcripts independently to improve coding quality. After merging the two separately-coded files, an interrater reliability Krippendorff’s c-alpha binary statistic was calculated at 0.80 using the inter-coder agreement function in ATLAS.ti. The RAs additionally prepared analytical memos of emerging themes from the transcripts. We grouped themes in the memos into three broad topics relating to habit facilitators, challenges, and modifications made by clients to improve ART adherence. As a third and final step, we extracted quotations to illustrate common themes or responses among ART clients. Each quotation was labeled with the client’s gender, age, and self-reported adherence status (i.e., missed any ART doses in last month or stopped ART for 2 or more days). Where possible, we also qualitatively examined similarities and differences in quotations by age and gender. The coding and analysis were conducted from February to August 2019.

## Results

### Sample characteristics

Table 1 describes the sample’s demographic and self-reported adherence characteristics. The mean age of ART clients enrolled in the qualitative study was 39, ranging from 19 to 63 years old (Table 1). Approximately two-thirds of the sample was female (69%, n = 29). The highest level of education for 43% of the clients (n = 18) was primary school followed by 31% (n = 13) who had completed secondary education. A small proportion of clients had vocational and/or university training (12%, n = 5). Unemployment was relatively high (36%, n = 15) compared to 52% (n = 22) of clients who were employed in the formal or informal sector. The mean number of years since HIV diagnosis was 11.5, ranging from 4 to 26 years. The majority of clients (81%, n = 34) reported having previously disclosed their HIV status to some or all of their close friends and relatives. Self-reported ART medication habits varied among the sample. Forty percent (40%, n = 17) of clients said they had missed an ART dose in the last month, and 21% (n = 9) reported stopping their ART regimen for 2 or more days. Almost two-thirds (62%, n = 26) of clients had undetectable viral load at the time of study enrollment.

**Table 1:** Demographic and adherence characteristics of study-enrolled ART clients (N = 42)

Variable	N (%)
Demographic characteristics (N = 42)	
Mean age (min–max)	39 (19–63)
Gender	
Male	13 (31%)
Female	29 (69%)
Highest level of education	
Primary	18 (43%)
Secondary	13 (31%)
University/vocational	5 (12%)
No education	1 (2%)
Missing^a^	5 (12%)
Marital status	
Single/never married	11 (26%)
Married	9 (21%)
Divorced/separated/widowed	17 (41%)
Missing^a^	5 (12%)
Employment	
Unemployed	15 (36%)
Employed (self/informal)	9 (21%)
Employed (formal)	13 (31%)
Missing^a^	5 (12%)
Adherence characteristics	
Mean years since HIV diagnosis (min–max)	11.5 (4-26)
Prior HIV disclosure to friends/family	
Yes, all	12 (29%)
Yes, some	22 (52%)
No	3 (7%)
Missing^a^	5 (12%)
Missed ART dose in last month	
Yes	17 (40%)
No	20 (48%)
Missing^a^	5 (12%)
Stopped ART for > 2 days	
Yes	9 (21%)
No	28 (67%)
Missing^a^	5 (12%)
Currently undetectable viral load	
Yes	26 (62%)
No	11 (26%)
Missing^a^	5 (12%)

^a^Demographic and adherence data collection at baseline was not possible for n = 5 clients

### Free-listing findings

Table 2 describes the frequency of reported habits from the free-listing exercise. A total of 47 distinct habits were mentioned across five categories. Habits that were mentioned by 10% or more of the sample (= 31 habits) are included in Table 2. The five most commonly reported habits were sleeping (85.7%), eating dinner (71.4%), eating breakfast (61.9%), traveling to school/work (61.9%), and bathing (61.9%) (Table 2). However, with the exception of bathing and eating dinner, fewer than half (< 50%) of clients mentioning these habits reported that they performed this habit at the same time or same location. Habits that were most commonly reported as being done at the same time were cooking a meal for a child (83.3%), having evening tea (71.4%), and bathing (69.1%), although not all clients mentioned doing these habits. Habits that were most commonly reported as being done at the same place were washing dishes (92.3%), praying in the morning (86.7%), cooking breakfast (83.3%), and bathing (82.8%). Interestingly, sleeping, which was mentioned by most clients (85.7%), was also reported by most as not occurring at the same time nor in the same place (63.9% and 61.1%, respectively). In contrast, taking ART medications, which was also mentioned by most clients (61.9%) had the highest reported percentage of occurring at the same time (92.3%). However, only slightly over half (57.7%) of clients who mentioned ART medication habits said they took their pills at the same location.

**Table 2:** Distribution of commonly reported free-list habits in rank order and with related time and location among study-enrolled ART clients (N = 42)

Summary of most commonly reported free-list habits^a^	Mentioned habit^b^ %	Performed at same time^c^ %	Performed at same location^c^ %
Eating habits (n = 9)			
Eating dinner	71.4	46.7	63.3
Eating breakfast	61.9	23.1	50.0
Cooking breakfast	42.9	44.4	83.3
Cooking dinner	33.3	28.6	57.1
Cooking lunch	28.6	41.7	41.7
Eating lunch	21.4	11.1	33.3
Going out to get food	16.7	42.9	42.9
Having evening tea	16.7	71.4	57.1
Cooking meal for child	14.3	83.3	66.7
Domestic habits (n = 7)			
Cleaning house interior	38.1	56.3	50.0
Washing clothes	31.0	38.5	53.9
Washing dishes	31.0	23.1	92.3
Gardening	28.6	50.0	58.3
Sweeping compound	23.8	50.0	60.0
Mopping compound	23.8	40.0	70.0
Tending to animals	14.3	50.0	66.7
Leisure habits (n = 7)			
Sleeping	85.7	36.1	38.9
Traveling to work/school	61.9	50.0	61.5
Watching TV	57.1	50.0	58.3
Praying in morning	35.7	66.7	86.7
Praying in evening	23.8	40.0	20.0
Visiting friends/family	21.4	44.4	22.2
Listening to radio/music	16.7	57.1	42.9
Hygienic habits (n = 6)			
Bathing self in morning	69.1	69.0	82.8
Bathing self in evening	64.3	37.0	82.8
Brushing teet	50.0	76.2	61.9
Washing face	33.3	50.0	100.0
Bathing child	28.6	50.0	58.3
Dressing self	16.7	71.4	57.1
Medication habits (n = 2)			
Taking meds in evening	61.9	92.3	57.7
Taking meds in morning	33.3	92.9	50.0

^a^List of free-list habits in each category that were reported by > 10% clients. Habits mentioned by < 10% are not presented

^b^Percentage of mentioned habits does not sum to 100% as responses were not mutually exclusive

^c^Calculated only for clients who previously mentioned relevant habit

### Qualitative findings from interviews

Table 3 summarizes the qualitative themes from interview discussions with ART clients. We identified several facilitators and barriers to habit formation of ART medications, including efforts among clients to modify their habits to improve ART pill-taking. These findings are summarized below with example labeled quotations.

**Table 3:** Summary of qualitative themes from interview discussions with study-enrolled ART clients (N = 42)

Topic	Emergent themes
Facilitators of ART medication habit	Linking ART medication-taking to existing routines
	Enlisting a person as a daily reminder
	Using alarms on other objects as a reminder
Challenges of ART medication habit	Unreliable time devices
	Irregular employment schedules
	Uncertainty of time or place to take ART medications
Modifications to habits to improve ART medication adherence	Packing or re-packaging ART medications
	Changing one’s leisure activities
	Carrying drinking water

## Facilitators of habit formation for ART medications

### Linking ART medications to existing routines

Several clients described developing a habit to take their ART medications by linking their ART medication time to other established routines, such a child’s non-HIV medication schedule or tea time. Stability in daily routines was often cited as a facilitator for ensuring ART medication adherence. For example, one client noted that she has a habit of providing medication to her daughter for another medical condition not related to HIV, and that this habit is tied to her habit of taking her own ART medications. A second client stated that he links taking his ART medications to the time he usually leaves the clinic, which he described as being a common routine. A third client described her habit of checking in first with her neighbors in the morning and then taking her ART medications each day after her morning tea. Radio and televised news programs were also reported to facilitate clients’ ART medication taking, as these programs aired at regularly scheduled times during the morning and evening hours.

*“I have my first born with sickle cells. When I give her medication, it reminds me that I have my own to take too.”*—Female, Age 28, Adherence Unknown

*“What reminds [me] is mostly what I do because I know I leave the clinic at 10:00am. So, I know that by this time, I have to be bathing. And, at 11:00am, I have to be taking my medication. Most of my time, I be at the clinic.”*—Male, Age 60, Missed ART dose last month

*“We are like three neighbors who wake up early. So, no one wants to wake up and leave without checking on each other. Because, there was a time [when] I was really sick, and everyone knew I was sick. They would wake up and ask me how my night was. And, we got used to it that way. In the morning, we check on each other just to see how we are all doing. So, I get that done. Then, I just know that the next thing is tea. Then, I take my septrine [ART medications] and go to work.”*—Female, Age 32, Missed ART dose last month

*“Sometimes I get reminded by the Muslims who pray at 8:00am. [And],..sometimes by the news [from the radio].”*—Male, Age 43, No missed ART dose last month

### Enlisting a person as a daily reminder

Some clients noted that asking their children (i.e., son, daughter, grandson) or another relative (i.e., mother, grandmother) to remind them has assisted in forming a habit of adhering to their ART medications. Another client said that he asked his mother to remind him if she notices that it is time to take his ART medications. He also described having a second person (a colleague) on whom he relies to be a cue. The colleague’s taking his medications reminded the ART client to also take his. However, it was not evident that having relatives and friends as reminders was a consistent habit given that clients described some relatives or friends as reminding them only if they also remembered or only in cases where the ART client was busy or sleeping. Clients also described motivating their enlisted person(s) by mentioning HIV-related illness and death due to poor ART adherence.

*“I have a grandson. If I am busy doing some things, he helps me check on the watch and tells me if it is time or if it’s about to [be time]. And [he] asks me, ‘Have you prepared your medication?’”* – Female, Age 47, Stopped ART for 2 or more days

*“I have a child who can wake me and says mom wake up and take the medicine. So, I ask her to check for the time, and if it’s 5* *min to time, I get to know that it’s now time to take the medication.”*—Female, Age 57, No missed ART dose last month

*“Sometimes I may dose off before taking the tabs. So, she calls me and tells me, ‘Your time for taking your medication has reached.’”*—Female, Age 26, No missed ART does last month.

*“…My son reminds a lot, because I told him that the only thing you can pay me for giving birth to you when you’re negative is to always remind me.”*—Female, Age 40, No missed ART dose last month

*“I have a daughter who knows I have to take medication, and my bag is always around. So, she tells me, ‘Mummy, you have to take your medication.’ She brings the bag to me since I told her that if she forgets to remind me to take my medication, I will die and leave her. I [also] have a phone on which I did set an alarm. And, even I remind myself as a person. …But sometimes my mother calls when it is time and asks, ‘That you? Are you done with taking your medication?’”*—Female, Age 26, No missed ART dose last month.

*“I have a grandson, who normally reminds me about the medicine and even my mother reminds me. If I am not around (like I am this side today), my brother or sister can call me and ask me whether I took the medicine. All on all, there are people who remind me that I haven’t yet taken the medicine. And the whole family knows, because I told that I am infected.”*—Female, Age 47, Stopped ART for 2 or more days

### Using alarms and other objects as reminders

A few clients mentioned using a cell phone alarm or watch alarm to remind them to take their ART medications. Sometimes alarms were coupled with other people who reminded them. While relying on an alarm was for some clients a challenge (due to it breaking or running out of battery charge), for others having an alarm on their cell phone was particularly helpful. In a few cases, clients described leaving objects around the house that would remind them, such as their “drug tins” or a glass of water. Clients also attributed having a “strong memory” to take ART medications as a result of valuable alarms and other cues.

*“I have a clock alarm that reminds me at around 10:00am. So, if I want to visit my people, I do it before the time. I also have my son who reminds me to take my medication. We call it ‘swallow.’”*—Male, Age 38, Adherence Unknown

*“I have an alarm. And, also, if my mum sees it’s time, she reminds me to take. There is also some Muslim who takes. So, I see him taking and take mine also because he takes his [medications] at around 9:30am. So, I calculate 30* *min and also take mine at 10:00am too.”*—Female, Age 28, Missed ART dose last month

“*I did set an alarm in my phone, and it reminds me every day both in the morning and evening. So, even if am doing something and the alarm rings, I just leave what it is and go take my medication.”*—Female, Age 38, No missed ART dose last month

*“I thank God for giving me a strong memory. I have my bottle of water and tin of medicine near my bed. So, every time I come back, I look at them and remember to take my medication. I can say I have never forgotten to take my medication any day.”*—Male, Age 57, No missed ART dose last month

## Challenges to habit formation for ART medications

### Unreliable time devices

Some clients described relying on their watch or cell phones to remind them to take their ART medications at the same time each day, and the challenge of doing this if their watch or cell phone was broken or away at a charging station. In these cases, clients reported asking someone for the time or sometimes taking their ART pills late by mistake. Clients lamented having unreliable time devices given that such devices were intended to prompt ART pill-taking, particularly in cases where they were likely to forget or in settings where they were likely to be distracted, such as at work or while traveling.

*“Most of the time when I am at work, I always be looking at the watch. And, when it clocks 9:00* *pm, I get to know that my time for taking the medication is about to reach. Even when my phone is messed up, I ask someone what the time is, so that I get to know how much time is left for me to soon take the medication.”*—Female, Age 33, Missed ART dose last month & Stopped ART for 2 or more days

*“I had my watch where I had put my alarm, but the alarm died. Now it’s in my mind. I have a phone that shows time. No alarm is on my phone…I use the phone to check time.”*—Female, Age 55, No missed ART dose last month

*“I can exceed and take [my medication] at 10:00am. There is nothing that stops me, but sometimes I might be busy with some things. And, by the time I check for the time, it might have exceeded. You see, in the village we charge with solar from a far place. And, sometimes they might give you the phone back [after] the whole day. So, you won’t be able to use the alarm. I might even spend two days without it [phone] if I took it for charging. And things like this happen.”*—Female, Age 47, Stopped ART for 2 or more days

### Irregular employment schedules

ART clients also discussed how developing medication habits was difficult given the irregularity of their employment responsibilities. For example, one client stated that he was able to take his ART medications while at home with little problem, but that it was hard to keep this ART medication routine when he was at work in another area away from home (referred to as “Bugiri”). In these cases, he reported having extended trips away from home without having carried his ART medications, due to limited time between travel assignments to re-stock or re-pack his ART medications. Another client attributed his ART medication adherence in the evening to consistently being at home, but often taking his morning tablets at different times due to being in route to his job. The unpredictability of this work travel undermined his efforts to form an ART medication habit at the same time each day.

“*Yeah, that’s what I was talking about. When I am home, I don’t do anything. But, when I am at work, we do a lot of work. So, I fail to get time. That’s why I told you the days I missed taking medicine. That’s why I told you in Bugiri that I did not take medication. I packed [the medications] for a short time. But, when you have just completed one job, they immediately send you to another job. So, there is no time to go home and get your medication. So, you just continue to the next job.”*—Female, Age 32, Missed ART doses last month & Stopped ART for 2 or more days

*“Sometimes I get to work from a different [way] or even get caught up at work, so I just take it from there. Mostly the morning tab[let]. …The one for evening I take it from home as a must. It’s only the morning medication [that is challenging], since I normally shift from one place to another. So, I take it from where the time of taking it has found me. I won’t lie to you. It changes.”*—Male, Age 48, Adherence Unknown

### Uncertainty of time or place to take ART medications

Clients also described being uncertain of when or how they would take their ART medications when their daily schedule varied from the norm. In these cases, they mentioned needing water or food (to avoid side effects or illness) and not having yet a plan for when or how they would take their ART medications at the preferred time, particularly if they were in public such as in a taxi or while attending a religious service. As two clients stated:

*“Like now I have come this side, so I don’t know whether I will take it from the taxis. I just have to be with water, then take it. So, I can’t tell you that I take it from the same place.”* – Female, Age 47, Stopped ART for 2 or more days

*“Yes, though on Sunday I sometimes take it to church. As long as I reach and prepare the class like at 8:30am or 8:45am. I rarely exceed 9:00am. That is when I take my medicine.”* – Female, Age 32, Missed ART dose last month

## Modifications to habits to improve ART medication adherence

### Packing or re-packaging ART medications (‘pocketing’)

One modification that clients mentioned to ensure adherence was to pack to their ART medications in a different container to take them while they were out and about, a practice commonly referred to as ‘pocketing of doses’. Other clients described additionally re-packaging their ART medications so as to avoid unintended disclosure. For example, one client described how she tried to take ART medications in hiding at her job–and removed her ART medications from the original packaging in order to take it with her to work without disclosure. She also mentioned taking ART medications sometimes at home to avoid suspicion from her employer. Another client described placing her ART medications in an envelope while at church given the expected delay in reaching home. A few clients also noted efforts to pack or re-package ART medications such that they were not damaged.

“*Yes, the only problem we have is some employers do not know we are sickly. So, we take the medication in hiding because they do not know because they sometimes discriminate. If it was not for the discrimination, we would be open and tell them at around this time, I have to take my medication. …That’s why most times I remove them from their original packs and put them in these polythene packs for tablets. And, even I seal it to prevent air from making them go bad*.”—Female, Age 32, Missed ART dose last month & Stopped ART for 2 or more days

*“I get my medicine and put it in an envelope. And, while at church I use a phone to check if it’s 9:00am. And, then I take the medication.”*—Female, Age 64, Stopped ART for 2 or more days

*“…I pack my medicine every day because sometimes I take it from my workplace.”*—Female, Age 38, Missed ART dose last month

*“I divide my medicine and take half away. So, what I am going to take, is what I put in this one since I can’t move all of it. Then the other much medicine [is put here] because they give me four bottles.”*—Male, Age 44, No missed ART dose last month

### Changing one’s leisure activities

Some clients said that they had changed aspects of their leisure activities to be more compatible with their ART medication schedule or physical reaction to their ART medications. For example, one client indicated that he had stopped drinking alcohol due to its counterindication with his ART medications as advised by the local clinic. Another client noted that she no longer went out to socialize with friends, such as going to a bar, as a result of concerns of mixing alcohol with her ART medications following clinic-based counseling. Returning home early from social outings to take one’s ART medications was also mentioned. By altering their schedules or coming home early, clients explained that these modifications ensured their medication taking.

*“Yes, even when I go somewhere, I make sure to come back early.”*—Female, Age 30, Missed ART dose last month

*“I even gave up on taking alcohol, because they taught us that it weakens the medicine*.”—Male, Age 60, Missed ART dose last month

*“I watch TV because I don’t go out since I gave up on those things, like going to the bar. Since I got to know that it tampers with my medication. So, I just stay home and am with my kids.”*—Female, Age 50, No missed ART dose last month

### Carrying drinking water

Carrying drinking water was also mentioned as a new habit to help support taking one’s ART medications at the appropriate time. Two clients noted that they packed water beforehand in cases where schedules were in flux. Carrying drinking water, for some, was considered a requisite to high ART adherence.

*“It is a must. If I go to church, and I know that I will delay, I take mineral water.”*—Female, Age 64, Stopped ART for 2 or more days

*“And I move with my medicine and water in the bag so if the alarm rings, I take it there and then.”*—Female, Age 38, No missed ART dose last month

## Discussion

All people living with HIV and on ART are faced with the daily demands of ensuring continued adherence. This qualitative study examined efforts to strengthen medication habits among clinic-enrolled Ugandan adults on ART for 2 or more years who had also demonstrated recent adherence problems, such as having missed an ART dose or refilled a prescription late. Our findings indicated that nearly all clients had one or more daily routines. Clients attributed successful ART adherence to linking medication-taking to other existing habits (i.e. meals, commuting, radio, prayers), having another person remind them, using alarms, changing their leisure activities, packing ART medications with water, or leaving pill tins/water in view. Such findings are consistent with evidence that behaviors prompted by situational cues are more frequently repeated, leading to habit formation [11–13]. However, incomplete adherence was attributed to having practices that did not always occur at the same time or place. Specifically, our study found that changes in employment hours, unpredictable travel schedules, and delays in social events, as well as device failures (i.e. phone, watches) made it difficult to maintain medication habits, suggesting that irregular practices do not facilitate repeated, subconscious behaviors.

These findings point to several lessons learned regarding the role of habit formation and ART use among treatment-mature clients. One lesson learned relates to clients’ efforts to link their ART regimen to daily habits they had already established. While clients described relying on several routines, the timing and frequency of these routines varied considerably. Clients often described routines that occurred most of the time or sometimes. In few cases did clients describe routines that were stable enough to remind them of their ART medications all of the time. For example, a child may remind his parent to take her ART pills, but the study did not find that this occurred consistently and without delay for all clients. Presumably, a person tasked with reminding an ART client would himself need to create the habit or remember. In fact, while clients in our study described daily routines as strengthening their ability to adhere to their ART medication regimen, they also appeared to be aware that relying on a single routine could lead to unintentionally missed doses. Our findings showed that a combination of strategies was often used to support ART medication habits and adherence. For example, clients referred to having someone to ask about their ART medications in addition to using an alarm or visual/audio cue. However, having this increase in “reminding” for long-term ART patients may reflect not only that taking their ART medications was not yet an automatic process, but also that their specific daily cues were unreliable in part. Changing schedules and changing environments weakened the predictability of medication and non-medication routines. In addition, we found that ART clients described multiple preparatory steps that they undertook to take their ART medications at the right time and place, such as arriving or leaving early, packing ART medications to carry out, bringing water, or preparing food. Challenges relating to habit formation for these prerequisite steps may also explain the ART adherence problems in this sample.

Interventions aimed at forming and maintaining habitual ART pill-taking behaviors may have the potential to improve adherence among long-term clients who unintentionally miss doses. On the one hand, specific advice for taking ART in changing and potentially unpredictable contexts may prove helpful for many clients. Our findings suggest that ART pill-taking occurred within a sequence of several routines, such as waking up, having tea, and then going to work. However, these sequences could vary in timing or completion, thereby disrupting pill-taking behaviors. Discussions with clients on how and why daily sequences change may help to identify ways to stabilize behaviors leading up to pill-taking times. On the other hand, providing habit formation advice that promotes taking ART medications in specific and unchanging contexts may also be beneficial. Other previously-published studies have shown that strong habits form when the target behavior is repeated in context of the same cue (i.e., taking pills immediately after morning tea or washing dinner plates) [11–14]. Our study found that clients with recent adherence problems were keen to utilize both time- and place-based cues to counter varying circumstances, but described fewer techniques used to stabilize these cues. For example, in some cases, the enlisted person to remind the client to take his or her ART medications was a child, which may have reflected clients’ beliefs that children had fewer daily distractions and were more committed to performing a repeated or simple task. However, questions remained regarding strategies implemented to support a child’s memory or habit to remind the ART client. Interventions that help clients to identify their most invariable activities and to develop strong associations between a constant routine and taking their ART medications may be needed. This might include helping clients who frequently forget a dose to self-examine the efficacy of their ART medication times and places and determine whether new times or places should be used. Habit formation intervention could also help clients to select times of day that are least likely to be affected by disruptions. A similar study on medication adherence among ART clients found that structured and routinized daily behaviors, in the form of habits, were positively associated with higher ART adherence [22]. In addition, a study in sexually-active adolescents found that habit formation with other HIV prevention technologies, such as prior condom use, was associated with higher future use [31]. In Uganda, most clients visit an ART clinic only once every 3 months, except at the start of their ART when visits are more frequent. Therefore, clinic-based counseling to support ART clients with known or suspected adherence problems may need to be supplemented with confidential home- or community-based habit-forming interventions.

Our findings also suggested that tailoring habit-forming interventions towards client demographic characteristics may be needed. For example, in this study, it appeared that younger clients were more likely to rely on their cell phones and radio/TV programs to remind them to take their ART medications. Therefore, future ART habit-forming interventions could leverage cell phone features, such as alarms or text messages, provided that airtime, battery, and repair issues are addressed [30]. Conversely, older clients, including those who were married or parenting, tended to rely more on their spouse or children for ART medication reminders. These enlisted persons could be more integrated in home-based habit-forming approaches. Gender-specific programming may also be relevant. As compared to women who reported multiple strategies, men more often reported relying on their own instincts or motivations to “remember” to take their ART medications. Studies among ART clients in sub-Saharan Africa have also shown that use of incentives, such as cash assistance, may improve medication adherence by intrinsically motivating self-perpetuating habits [32, 33]. Habit formation guidance that takes into account individual preferences for various types of ART medication cues (i.e., digital, financial, social, intrinsic, external, home, community, or clinical) may be an important part of ensuring continued adherence in long-term ART clients.

### Limitations and strengths

The study’s limitations should be taken into account. First, recruitment was limited to clinic-enrolled adults who had recently joined a randomized clinical trial designed for clients who had demonstrated recent adherence problems. As a result, clients may have been biased towards a less adherent sample than expected in the general population of adults on ART. In contrast, it is plausible also that clients may have been biased towards a more adherent sample than expected among people living with HIV who were lost to ART follow-up or not enrolled in a study. Interviews were also limited to treatment-mature ART clients only, rather than staff and household members providing adherence support. Clients may have had fewer or varying insights into their own habit formation processes for ART than observing friends and family. They may also have been inclined to report predominately positive experiences rather than dwell on missed doses. Another limitation of the study was that no causal associations can be made given the cross-sectional and qualitative nature of the study. For example, adherence problems among clients may be due to intentional reasons not linked to habit formation. Consideration of non-conscious habits along with more conscious motivations (i.e. medication beliefs) could yield further insights for effective adherence interventions. Finally, the small sample size, although common in qualitative studies, was also a limitation, as it is possible that the sample was not sufficiently large enough to identify differences in ART medication habits between sub-groups. As each client was interviewed only once, we were additionally unable to obtain reflections from clients over time as they moved in and out of non-adherence. However, the strengths of this study were the inclusion of individuals on long-term ART with recent adherence problems, use of mixed qualitative methods in a sub-Saharan African clinical setting, including a rapid free-listing approach to quantify behaviors, and investigation of the relatively novel concept of habits relative to HIV-related antiretroviral therapies.

## Conclusion

Prior research suggests that individuals receiving ART for several years commonly fail to take all of their pills over time, increasing their risk to HIV-related morbidity and mortality. Forming habits to take ART medications repeatedly and without conscious effort may support high long-term adherence in treatment-mature clients with declining motivation over time. Our study found that despite having taken ART for several years, clients relied on multiple ART medication reminders with limited consistency and reliability. More research is needed on the role of habits for long-term ART adherence and on the design and implementation of home-, community-, or clinic-based habit-forming interventions that are tailored to ART clients’ existing routines.
